# Dynamic behavior of metabolic syndrome progression: a comprehensive systematic review on recent discoveries

**DOI:** 10.1186/s12902-021-00716-7

**Published:** 2021-03-22

**Authors:** Pezhman Bagheri, Davood Khalili, Mozhgan Seif, Abbas Rezaianzadeh

**Affiliations:** 1 Student research committee, Shiraz University of Medical Sciences, Shiraz, Iran; 2 Prevention of Metabolic Disorders Research Center, Research Institute for Endocrine Sciences, Shahid Beheshti University of Medical Sciences, Tehran, Iran; 3 Department of Biostatistics and Epidemiology, Research Institute for Endocrine Sciences, Shahid Beheshti University of Medical Sciences, Tehran, Iran; 4 Department of Epidemiology, School of Health, Shiraz University of Medical Sciences, Shiraz, Iran; 5 Colorectal research center, Shiraz University of Medical Sciences, Shiraz, Iran

**Keywords:** Metabolic syndrome, Natural history, Disease progression, Dynamics

## Abstract

**Background:**

The assessment of the natural history of metabolic syndrome (MetS) has an important role in clarifying the pathways of this disorder.

**Objective:**

This study purposed to provide a rational statistical view of MetS progression pathway.

**Methods:**

We performed a systematic review in accordance with the PRISMA Statement until September 2019 in the Medline/PubMed, Scopus, Embase, Web of Science and Google Scholar databases. From the 68 found studies, 12 studies were eligible for review finally.

**Results:**

The selected studies were divided in 2 groups with Markovian and non-Markovian approach. With the Markov approach, the most important trigger for the MetS chain was dyslipidemia with overweight/obesity in the under-50 and with hypertension in the over-50 age group, where overweight/obesity was more important in women and hypertension in men. In non-Markov approach, the most common trigger was hypertension. Transition probability (TP) from no component to MetS were higher in all Markovian studies in men than in women. In the Markovians the combination of dyslipidemia with overweight/obesity and in non-Markovians, hyperglycemia with overweight/obesity were the most common combinations. Finally, the most important components, which predict the MetS, were 2-component states and hyperglycemia in Markovian approach and overweight/obesity in non-Markovians.

**Conclusions:**

Among the components of the MetS, dyslipidemia and hypertension seems to be the main developer components in natural history of the MetS. Also, in this chain, the most likely combination over time that determines the future status of people seems to be the combination of dyslipidemia with obesity or hyperglycemia. However, more research is needed.

**Supplementary Information:**

The online version contains supplementary material available at 10.1186/s12902-021-00716-7.

## Background

Metabolic syndrome (MetS) is defined as a coexistence of clusters of metabolic disorders and cardiovascular risk factors including central obesity, hypertension, hyperglycemia, high triglyceride, and low HDL (high-density lipoprotein) cholesterol levels [1]. Various international studies have demonstrated the association of this disorder as one of the major health challenges of the century that has attracted the attention of many scientists [2–4], with the occurrence of cardiovascular disease (CVD), type2 diabetes, and cancer, as well as the occurrence of death from heart disease and all-cause mortality [5–8]. The disorder prevalence estimated 25% in developed countries [9, 10].

Recent studies on MetS have focused on the development of concepts and definitions and epidemiological and etiological research [11–15] as well as definition criteria and there are less studies on the natural history of this disorder [16]. The meaning of the natural history of MetS here is the process of combining the components of the MetS together and creating disorder state in individuals during the time. On the other hand, these rare studies on the natural history of MetS have also given conflicting information [16–21] about the first component that appears in a person or trigger, the probabilities of transition between components and states, the prediction of subsequent states throughout the chain and so on, that in the next sections, are presented and compared in detail.

**Fig. 1:**
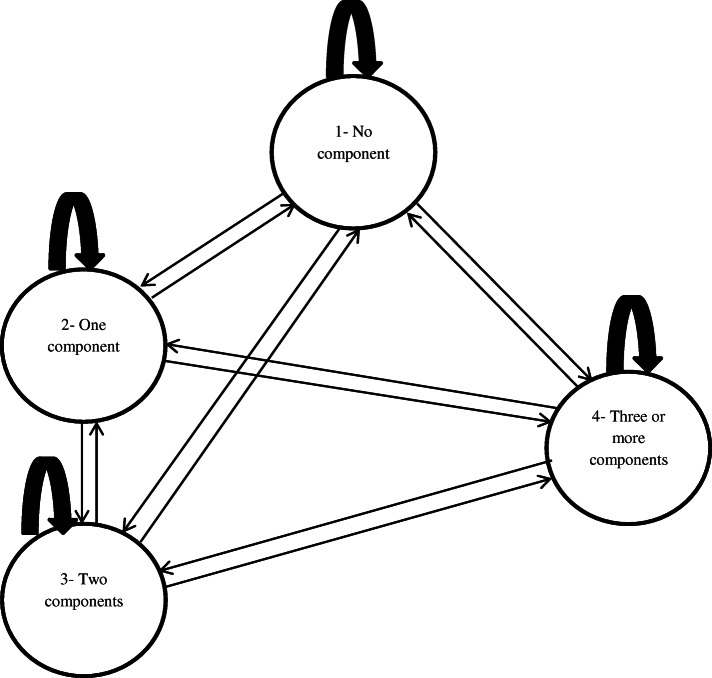
Hypothetical dynamicity of MetS natural history

There are different criteria for the diagnosis of MetS [15, 22, 23]. These criteria are mainly based on having at least several components of central obesity, hypertension, hyperglycemia, high triglyceride, and low HDL cholesterol (In most sources, the combination of high triglyceride and low HDL has been described as dyslipidemia) concurrently. Depending on the number of components that a person can give at each point in time, various scenarios for states of the disorder may develop over time progress. In a study [19], a 12-state classification (one state without components, four states of isolated components, six states of a 2-component combination, and one state of MetS) was specified. In another study [21], a 8-state (no component, five isolated component states, 2-component state, and MetS state) was designated. Also in others [16, 24], a 7-state classification (no components, isolated hypertension, isolated overweight/obesity, isolated hyperglycemia, isolated dyslipidemia, a 2 component state, and the MetS state) was presented. The having of each of these states in individuals is completely dependent on their lifestyle and occur dynamically and randomly during the disorder establishment; So that, there is possibility of transitioning from one state to another (disease progression), backward (disease recovery), stationary in one state (stopping in one state) or transverse transitions (substituting one component for another). This dynamic and random occurrence and transitions between different states somewhat complicate the natural history of this disorder and its study [19]. The following figure (Fig. 1) attempts to show some of these dynamics and complexity.

The progression of the MetS is similar to a process in which components appear sequentially. It is commonly believed that the first component that appears in a person with a high incidence is the primary trigger of the chain leading to the MetS. Scientists also believe that in the MetS prediction process, the component that results in the highest incidence of this disorder plays an important role in the development of the MetS [19]. Of course, this is still a controversial issue today [21, 25, 26]. What is clear is that assessing the dynamics of the MetS progression process has an important role in clarifying the pathways of this disorder. Although the exact pathogenesis of this disorder is still unclear, since components in MetS occur together as a set, it can be said there are common mechanisms for the MetS that are widely believed by researchers to play a central role in insulin resistance in this process [19, 27]. In spite of the evidence, it is still unclear and contradictory issues in this longitudinal path that include the main trigger of the chain leading to MetS, the probabilities of transition between states, the probability of stopping in one state, the prediction of subsequent states throughout the chain, the probability of the simultaneous occurrence of the most common component or combination of components, also the most common component of the MetS progression process trigger and the most important transition between the MetS progression process states. Therefore, the review of the existing evidence is necessary to get a comprehensive picture of the natural history of this disorder and provide a rational view of its progression mechanism.

## Methods

This systematic review was performed based on the PRISMA (Preferred Reporting Items for Systematic Reviews and Meta-Analyses) Statement [28]. The PICO/ PECO (population, intervention/exposure, comparison, and outcome) criteria and the research question used to determine keywords and formulate a search strategy are shown in Table 1.

**Table 1:** PICO/PECO criteria description and research question defined to systematic review

Parameter	Description
Population	Individuals with various components of MetS
Indicator	Metabolic syndrome progression
Comparison	Various components with each other
Outcome	Dynamic behavior of the natural history of the disease
Research question	How is the dynamic behavior of the MetS natural history progression?

### Search strategy

The timeframe for a systematic search of resources was set until 31 December 2019. Studies published in the Medline/PubMed, Scopus, Embase, Web of Science and Google Scholar databases were searched using a combination of the following strategies and Boolean operators with parenthesis and quotation marks:

1. (((((((((Metabolic syndrome [Title/Abstract]) OR (Metabolic X Syndrome [Title/Abstract])) OR (Metabolic Cardiovascular Syndrome [Title/Abstract])) OR (Syndrome X [Title/Abstract])) OR (MetS [Title/Abstract])) AND (Development [Title/Abstract]))) OR (Progression [Title/Abstract])) OR (Trajectory [Title/Abstract])) OR (evolution [Title/Abstract])
2. ((((((((((((Metabolic syndrome [Title/Abstract]) OR (Metabolic X Syndrome [Title/Abstract])) OR (Metabolic Cardiovascular Syndrome [Title/Abstract])) OR (Syndrome X [Title/Abstract])) OR (MetS [Title/Abstract]))) AND (Pathway [Title/Abstract])) OR (Mechanism [Title/Abstract])) OR (Natural history of disease [Title/Abstract])
3. ((((((((((((((Metabolic syndrome [Title/Abstract]) OR (Metabolic X Syndrome [Title/Abstract])) OR (Metabolic Cardiovascular Syndrome [Title/Abstract])) OR (Syndrome X [Title/Abstract])) OR (MetS [Title/Abstract])))) AND (Dynamic behavior [Title/Abstract])) OR (Dynamic development [Title/Abstract])

The search results were imported into EndNote® version X7 software (Thomson Reuters, New York, USA) in. enl format and any duplicate studies were automatically deleted. All references cited in the articles were also searched to screen for studies not found in the main search strategy.

### Selection criteria

All articles related to the research question that included a clear description of the “progression of the MetS and its components” [24] and the longitudinal pathways for the progression of the MetS and its components in title or abstract were included in this study. There were no initial time points for the search. But only English language studies were searched. In the first stage, the titles and abstracts of all obtained studies were screened and ineligible articles were excluded from the study process. The full text of the remaining articles was then carefully evaluated. Posters and conference papers and articles containing inadequate information on the checklist items listed in the next section were excluded from the review process.

### Critical appraisal

The appropriateness of all the obtained articles was assessed independently by two reviewers (H.A. and M.GH.) using the Newcastle Ottawa Scale [29]. The purpose of the critical evaluation was to examine the methodological quality of the articles and the noncompliance with standards in the design, procedure, and analysis. Disagreements were resolved by discussion with a third reviewer (P.B).

### Data extraction

To required information extraction, a designed checklist consisting of author, study design, year of publishing, sample size, location as well as information on the TP between the states mentioned in the preceding section, along with their predicted values, the criteria used to define the MetS, the follow-up period, the statistical model, and the main chain trigger leading to MetS separately sorted by demographic variables (age and sex) was used.

## Results

**Fig. 2:**
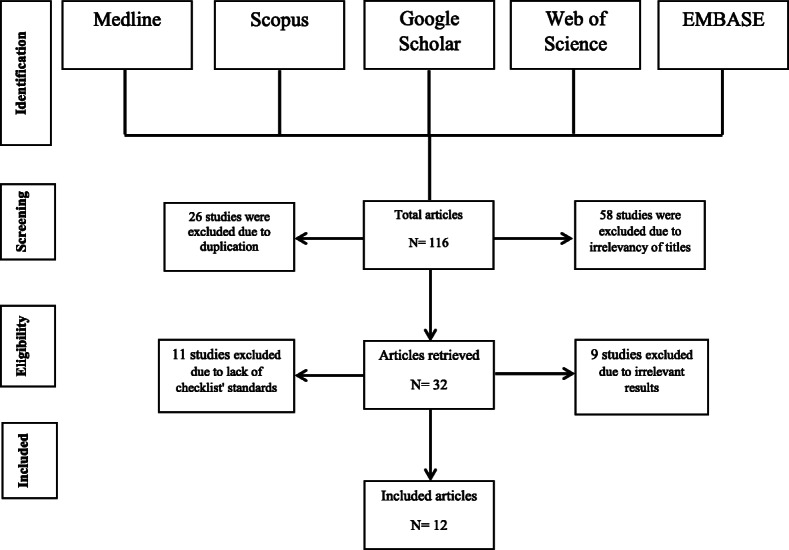
Flowchart of systematic reviews included in the final analysis

The literature searches process description:

The process of searching and selecting related articles shown in Fig. 2. Initial searches identified 116 articles. Of these, in a title-based screening, 84 studies were excluded from the review process for reasons of duplication and irrelevancy of titles. During the quality control phase, from 32 remaining article, 20 articles were removed due to lack of information in results such as outcome data and main results, weak design, setting and statistical method. Finally, the 12 articles entered in the main study process.

The general information of included articles:

The general information of the selected studies is presented in Table 2. Based on the statistical models used in these studies, overall, they can be divided into two categories of articles with Markovian model (4 studies) and articles with other statistical models (8 studies) which were mainly regression models. Markovian articles with a total sample size of 37,415 were conducted, mainly (75%) in China [16, 19, 24] and in 2018 (50%) [19, 24]; whereas most of the non-Markovian articles with a total sample size of 31,631 conducted before 2013 and mostly in European countries except one [26] that in terms of time conducted in 2017. Among the Markovian studies, the Chinese Diabetes Society (CDS) definition criterion was used to define MetS in most articles (3 from 4 study), while the most common definition criterion among non-Markovian studies was the NCEP (National Cholesterol Education Program). The mean follow-up time between the Markovian and non-Markovian articles was 6 and 7.3 years respectively.

**Table 2:** Basic information of selected articles

First author	Year of publishing	Location	Sample size	Study design	Definition criteria for MetS	Follow up/study time (year)	Statistical model
Xiaoxian Jia [19]	2018	Dongying City/china	21,777	longitudinal study	Chinese Diabetes Society (CDS)	6	Markov modeling
Xiaoxiao Chen [16]	2014	Dongying City/china	7510	longitudinal study	Chinese Diabetes Society (CDS)	6	Markov modeling
Xiao Tang [24]	2018	Dalian City/china	5881	longitudinal study	Chinese Diabetes Society (CDS)	7	Markov modeling
Lee-Ching Hwang [21]	2013	Taiwan	2247	longitudinal study	revised AHA/NHLBI	5	Markov modeling
Maria A. Barcelo [26]	2017	Catalonia, Spain	13,030	retrospective cohort	the American Diabetes Association	8	multivariate survival analysis
Bernard M. Y. Cheung [18]	2008	Hong Kong/china	1944	longitudinal study	NCEP and IDF	6	Multivariate logistic regression
Angelo Scuteri [17]	2009	Baltimore/ Maryland	967	longitudinal study	NCEP	6	Linear mixed-effects regression models
Angela D. Liese [14]	1997	U.S.	6113	retrospective cohort	–	6	Multiple logistic regression
Pratik A. Patel [30]	2013	Minnesota	1042	longitudinal study	NCEP	8.3	Cox regression analysis
Oscar H. Franco [31]	2009	Framingham	3078	longitudinal study	NCEP	9	logistic regression/ Cox regression analysis
Robin Haring [25]	2012	Pomerania /Central Europe	3187	longitudinal study	NCEP	5	Network-Based Approach
John A Morrison [32]	2005	Ohio	2270	longitudinal study	NCEP	10	Longitudinal regression models

**Fig. 3:**
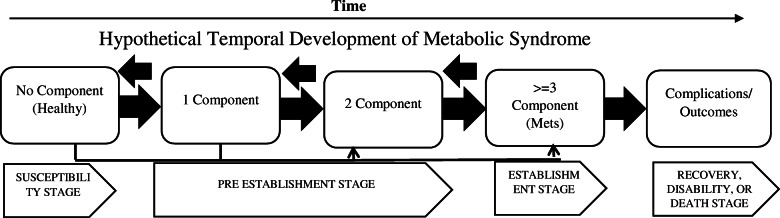
hypothetical temporal development of the metabolic syndrome

The exclusive information of included articles:

1. Markovian Studies:

Based on the findings, we are faced with stages that are the process of the establishment of this disorder over time and illustrate the natural history of the MetS (Fig. 3). In this figure, the interconnected relationships between factors and random changes over time alongside the simultaneous occurrence of some components make it difficult to study the natural history. This issue was implicitly suggested as the most important justification for using the Markovian model, which pays special attention to random variations in random processes, to study the natural history of the disorder in the studies. A continuous Markov process is a random model for describing a sequence of possible events in which the probability of each event depends only on the previous state [33]. Many chronic diseases progress naturally. Multistate Markov models are used to describe the progression in which a person follows a series of states as the disease progresses [34]. According to Xiaoxian et al. [19], at each stage of the transition, individuals can stay in their state or transition to one of the 11 other states. Therefore, overall, a total of 144 transitions can be considered for this dynamic process.

Since these four studies were all conducted with one statistical model, they had a similar structure in presenting their results. The results of this group of studies on the population aged 18–88 years included two parts of calculating the TP between states of the natural history of MetS and predicting its progression pattern using simulation. In the first section, based on the TP calculated between states, the most important chain trigger leading to the MetS, the most common and frequent transitions, as well as the most common combination between components in the MetS establishment process were mentioned. In the second part, based on the simulations performed over a given time period, the most important component that had progressed to a full establishment of the MetS was identified.

Results of section one - TP and related issues:

Accordingly, dyslipidemia with overweight/obesity, especially in the under-50 age group, and hypertension, especially in the over-50 age group, have been identified as the most important triggers of the natural history of MetS chain [16, 19, 21, 24]. One study [21] also pointed to the higher importance of overweight/obesity in women and hypertension in men as the primary trigger. In all of these studies, the prevalence of all components, including hypertension, dyslipidemia, hyperglycemia, overweight/obesity, and MetS, over time and with age increasing relative to baseline mentioned. In only one study [19], the prevalence of overweight/obesity and dyslipidemia in men did not show an increasing trend with age. In all four studies, the TP of no component or health toward MetS was higher in men in all age groups than in women, although the gap decreased gradually with age. However, in one study [21] in women, the transition from no component to overweight/obesity and low HDL states was reported higher than men. Women were also more likely to transition from overweight/obesity to MetS after the age of 50 [16]. Interestingly, the probabilities of reverse transitions from each of the states to the no component state were lower in men than in women. However, the overall probability of reverse transitions from any state to no component state decreased significantly with age in both genders [24]. Also, in this study, among all states, the transition from isolated overweight/obesity state to no component state was less likely than reverse transitions to other states. Also, the TP from any of the isolated states to the MetS at an older age was significantly higher than earlier age [24].

Describe TP tables:

Tables of distribution of the TP between different states are presented in (Additional file 1: Tables 7–18). Due to the differences in age and sex groupings between the articles in this group and the high extent of the tables, it was not possible to accurately compare the TP between studies or to summarize the results. Therefore, in general, the results of the studies were determined in terms of the highest and lowest values of TP between states in general, of the four studies of high and low values without regard to the state (Table 3), and separately by each of the states (Table 4), which set the highest and lowest values in each of the states, was summarized. In other words, among the results of the four studies, the highest and lowest values were selected and presented in general, and in each case. In Tables 3 and 4, values in the cross cells for each component were not considered. In general, the highest TP were dedicated to dyslipidemia to no-component state and hyperglycemia and MetS both toward the 2-component state that occurred in men, again men and women respectively.

**Table 3:** The largest and smallest probability values among all the states in the general case

	To any state	
	Max (%)	Min (%)
No component	13.33 to dyslipidemia^b^	.2 to hyperglycemia^a^
Overweight or obesity	37.81 to no component^b^	0^c^
hypertension	32.76 to any 2-component^a^	0^c^
dyslipidemia	55.11 to no component^a^	0^c^
hyperglycemia	43.75 to any 2-component^a^	0^c^
2-component	31.49 to MS^a^	0^c^
≥3 component (MS)	42.31 to 2-component^b^	0^c^

^a^in male/^b^in female/^c^ to multiple states in both gender

**Table 4:** The largest and smallest probability values by each state

	No component		Overweight or obesity		hypertension		dyslipidemia		hyperglycemia		2-component		≥3 component (MetS)	
	Max (%)	Min (%)	Max (%)	Min (%)	Max (%)	Min (%)	Max (%)	Min (%)	Max (%)	Min (%)	Max (%)	Min (%)	Max (%)	Min (%)
No component			10.15^a^	.91^a^	12.11^a^	.41^b^	13.33^b^	.81^b^	11.24^a^	.16^b^	8.33^b^	.42^b^	3.09^a^	.41^b^
overweight or obesity	37.81^b^	2.16^a^			3.23^b^	0^c^	5.38^b^	0^c^	2.46^b^	0^c^	28.75^a^	3.41^b^	11.76^b^	1.25^b^
hypertension	24.26^b^	0‡	8.48^a^	0^c^			3.96^b^	0^c^	2.42^a^	0^c^	32.76^b^	3.38^a^	13.79^b^	.79^a^
dyslipidemia	55.11^b^	7.91^b^	7.14^b^	0^c^	9.52^b^	0^c^			4.76^b^	0^c^	29.73^a^	1.43^b^	8.63^a^	0^c^
hyperglycemia	23.28	0^c^	5.19^b^	0^c^	6.9^a^	0^c^	6.9^a^	0^c^			43.75^a^	3.1^b^	30.77^b^	0^c^
2-component	24.68^b^	0^c^	21.56^a^	0^c^	26.15^a^	0^c^	12.99^a^	0^c^	25.91^b^	0^c^			31.49^a^	4.21^b^
≥3 component (MS)	14.29^b^	0^c^	10^a^	0^c^	13.52^b^	.45^b^	4.76^b^	0^c^	3.9^b^	0^c^	42.31^b^	2.71^b^		

^a^male/^b^female/^c^ multiple in both gender

Separately, the most likely transition occurred from no component to dyslipidemia, from overweight/obesity to no component, from hypertension to each of the 2-component states, from dyslipidemia toward the no component, from hyperglycemia to each of the 2-component states, from each of the 2-component states to the MetS, and eventually from the MetS state to each of the 2-component states (Table 4).

The lowest TP were also occurred from no component to hyperglycemia, from overweight/obesity to hyperglycemia, dyslipidemia, and hypertension, from hypertension to any of the model states except 2-component and MetS, from dyslipidemia to all states except no component and 2-component, from hyperglycemia to any model states except 2-component and MetS, from each 2-component states to all states except MetS and finally from MetS to all states except hypertension and 2-component states. Overall, the highest TP was related to the transition from dyslipidemia to no component. Secondly, the TP to the 2-component state was higher than in other states.

Results of section two - Risk prediction of the development of MetS:

This section describes the Risk prediction process of MetS over a period of at least 10 years using simulation techniques. In risk prediction process that performed with Markov modelling using TreeAge Pro 2011 (Tree Age Software, Inc., Williamstown, MA), the mean value of annualized transitional probabilities used to predict the effect of different initial states on the development of MetS in the future in every age and gender group. The commonality of all these studies was the increasing trend of progression of the MetS over time. In all 4 studies in this group, the development of MetS was higher in individuals with either 1-component or 2-component, especially 2-component states, than in the no-component group. Overall, the trend of developing the MetS overtime was higher in men than in women.

Overall, in the two states, the two studies reported the superiority of the TP from any of the 2-component states, especially those involving the overweight/obesity component to the MetS [21, 24]. Two other studies [16, 19] also indicated the superiority of hyperglycemia or 2-component states including hyperglycemia in the time horizon of study (Table 5). In other words, the most common states in the study simulation process were 2-component states and hyperglycemia. Other information in this group is presented in Table 5.

2) Non- Markovian studies:

**Table 5:** Specialized information on articles with Markovian method

First author	The most likely trigger of MetS by age or sex groups	Age groups	The most common transition	The most common combination	The most likely component to develop MetS
Xiaoxian Jia [19]	dyslipidemia or overweight/obesity in 18–49	18–88	isolated state of overweight/obesity, hypertension, or hyperglycemia to dyslipidemia	Dyslipidemia + overweight/obesity before age 50	isolated Hyperglycemia or overweight/obesity + hyperglycemia in men in all age groups
	Dyslipidemia or hypertension in over 50			Dyslipidemia + hypertension after age 50	isolated Hyperglycemia or hypertension + hyperglycemia in women in all age groups
Xiaoxiao Chen [16]	Men under 60 years old and women under 50 = overweight or obesity state and dyslipidemia	≥18	from any isolated state to the 2-component state	unclear	Isolated hyperglycemia overall
	Hypertension in over 60 in males and over 50 in females				
Xiao Tang [24]	In men under 40 years old and in women obesity and dyslipidemia	20–60	unclear	unclear	2-component in men and women
	In men over 40 years old dyslipidemia and hypertension				
Lee-Ching Hwang [21]	In women, abdominal obesity or low HDL	18–45	unclear	unclear	2-component in men
	In men, high BP or a 2-component state				Isolated obesity in women

As seen in Table 1, all of the studies in this group were conducted prospectively with longitudinal approach. Most of these studies have focused on the trend changes in the prevalence or incidence of MetS (development of MetS) and its association effect size on components, along with the frequency of component changes over time. In none of them, according to the used statistical model, there was no hint of a TP. The age range of the individuals in this group was between 9 and 80 years. The common feature between the exclusive information of Markovian and non-Markovian articles was the most common component, naturally known as a trigger, and the most important component predicting MetS in the future. Accordingly, with the non-Markovian approach, the most common component initiating the MetS development process was hypertension and the most important predictor of the MetS in the future, was overweight/obesity. In some studies, [14, 26, 31] the greater role of hyperglycemia in the development of the MetS has been pointed out. However, in the Barcello study [26], the higher importance of hyperglycemia with hypertension was emphasized in the earlier onset of the MetS episode. Other studies, such as the Oscar study [31], have emphasized the dual importance of combining the three components of hypertension, hyperglycemia and obesity/overweight in predicting the risk of the MetS and the speed of its development, as well as the mortality of its consequences, such as cardiovascular events and the importance of the components related to overweight/obesity and dyslipidemia states in the speed of development of the MetS has not been much emphasized. Regarding the absence of MetS during the study period, only two studies indicated that 61% of the total [26] and elsewhere 66% of men and 76% of women [17] with one or two components had no MetS and were undergoing a steady or reverse trend toward recovery.

Another important issue in these studies was the most important and most common combination of components. In two studies, the combination of overweight/obesity, hypertension and hyperglycemia [30, 31], in another study the combination of hypertension and overweight/obesity [25] and in one study the combination of all four components [32] were introduced as the most common combination. In terms of the composite components increasing in the natural history process, only one study [31] indicated that hyperglycemia and overweight/obesity experienced the highest increase. Also, in terms of the contribution of each component overtime for getting MetS, only one study [26] indicated that 55.7% of people with hyperglycemia, 49.4% of people with hypertension, 40.2% of patients with dyslipidemia and 42.2% of obese/overweight patients had MetS during the eight-year follow-up period. Other information in this group is presented in Table 6.

**Table 6:** Specialized information on articles with the non-Markovian method

First author	Age groups/mean age	The most frequent component	Main predicted factors for MetS development	The most likely component to develop MetS	Developed MetS%
Maria A. Barcelo [26]	≥15	overweight/obesity	smoking and alcohol, age over 75 and male/ Triglycerides, obesity and low HDL	overweight/obesity and low HDL	39
Bernard M. Y. Cheung [18]	52 ± 12	Low HDL	Age by NCEP/ Systolic blood pressure/ FPG in men Triglycerides and HDL	overweight/obesity	21·9 and 14·3 per 1000 person-years by the NCEP and IDF criteria
Angelo Scuteri [17]	52.4 ± 17.5	elevated blood pressure	Low HDL and high BP and Waist circumference	overweight/obesity or triglycerides and lower HDL	25.5% in men and 14.8% in women
Angela D. Liese [14]	53.5 ± 5.6	unclear	High fasting insulin/ BMl, and WHR/ sex/ ethnicity	high insulin/ BMI > 30 / high waist-to-hip ratio	unclear
Pratik A. Patel [30]	> 45	elevated blood pressure	unclear	HTN/ obesity/ diabetes mellitus	unclear
Oscar H. Franco [31]	51.6	elevated blood pressure	unclear	overweight/obesity	10.6
Robin Haring [25]	20–79	hypertension	hypertension and overweight/obesity as well as their combination/ Low HDL	unclear	42.4
John A Morrison [32]	9–19	HDL-C	waist circumference/ triglyceride/ BMI	waist circumference	3.5% in black girls and 2.4% in white girls

## Discussion

The results of this study showed that with the Markov model approach, the MetS in its developmental process has the capability of a total of 144 bidirectional transitions in twelve states. With the Markov approach, the most important trigger for the MetS chain was dyslipidemia with overweight/obesity in the under-50 age group, and dyslipidemia with hypertension in the over-50 age group. Also, with the non-Markov approach, the most common component of the MetS initiation process was hypertension.

**Fig. 4:**
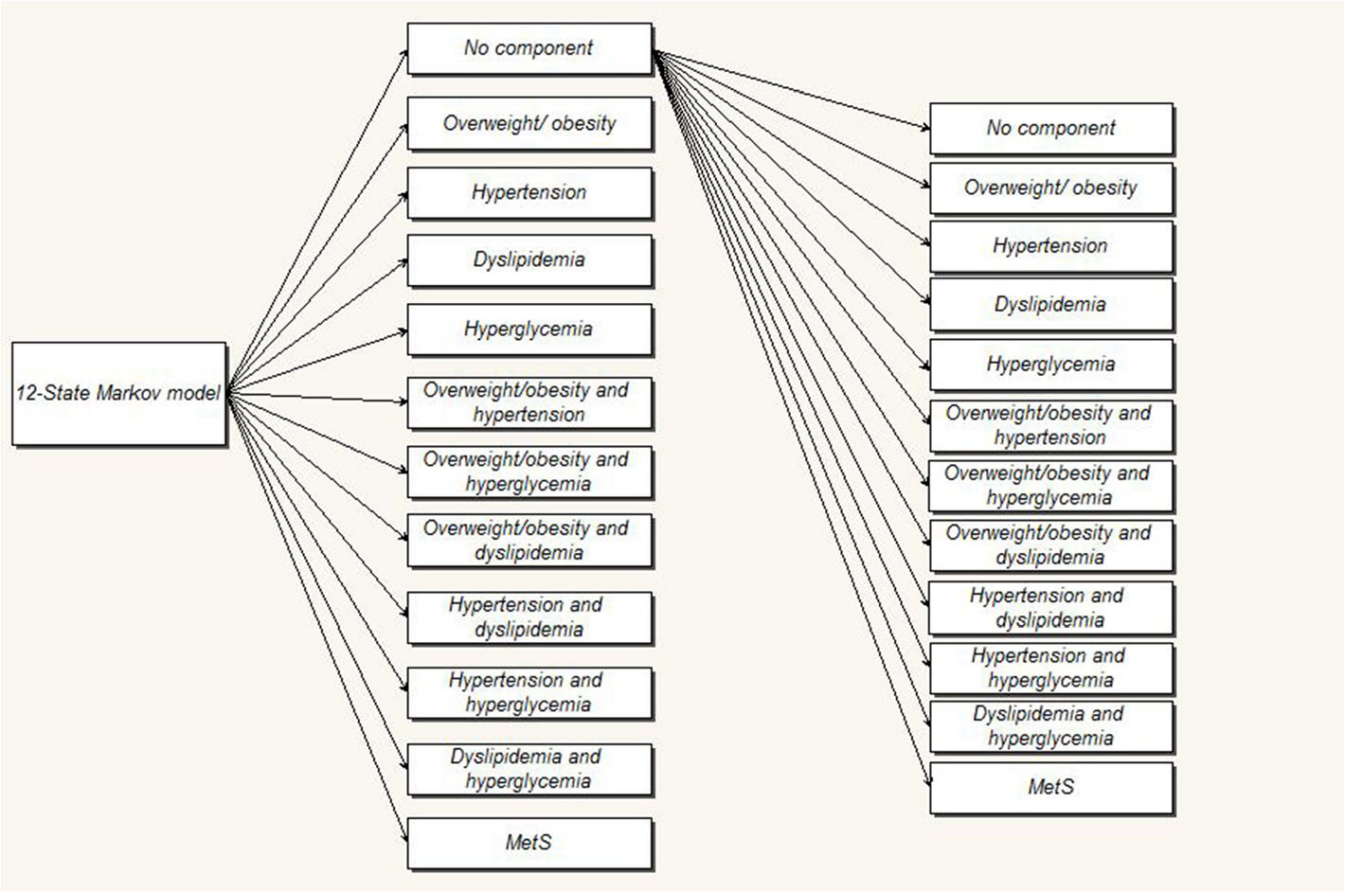
a 12-state Markov model used to describe the progression of MetS states

In the studies with the Markovian approach [16, 19, 21, 24], as mentioned earlier in the introduction, different models of 7, 8 and 12-states has been used. But the most common state-model used in these studies was the 7-state model, which does not mention the 2-component and dyslipidemia states separately and in detail in comparison to the 8- and 12-state modes. Yet, the 12-state model has more details. In a reversible Markov model, all possible transitions from one state to another are possible and illustrated (Fig. 4). All subjects can be in one of 7, 8, or 12 state-models at the beginning of the study. Then, in the next period that usually considered one year later in these studies, one can stay in or transition to any other state. An important advantage of the Markov model is the division of the disease development process into several states and the possibility to simulate this process based on the characteristic of the disease transmission capacity from one stage to another [19, 35, 36].

It is believed that the first component with the highest incidence is the main trigger of the MetS development process and this is a basis for trigger determination [19]. In non-Markovian models, the basis for trigger determination is not longitudinal and based on the predictive power of the components, their role and importance are judged. As shown in Table 5, the central core of triggers in different Markovian studies, which is essentially a controversial issue [21, 25, 26], is the set of Dyslipidemia, overweight/obesity and hypertension components. In all of the studies listed in Table 5, these components are present at almost all levels of study. Of course, the reason for the difference between the various studies is that its dependency on demographic characteristics such as age, sex, or other characteristics; as if, Xiaoxian [19] also refers to this issue. Of course, there is also a marked difference between the results of the Markov and non-Markov studies, which may be due to the difference in the MetS definition criteria of the Markov and Non-Markov studies. Because the criteria used in non-Markovian studies is a global criteria such as IDF (international diabetes federation), NCEP, etc. While in three Markovian studies of four studies, the CDS local criterion was used (Table 2). In our study, in both Markovian and non-Markovian approaches, the prevalence of all components in both sexes was increasing with age and with time. In only one study with Markov’s approach, the prevalence of overweight/obesity and dyslipidemia in men didn’t increase with age. According to the available evidences, prevalence of the Mets increases with age due to endogenous sex hormone levels changes [37, 38]. In women for example, Wu et.al [38] indicate that if age at menopause is greater than 49 years, an increase in years since menopause confers a negative influence on glucose tolerance that is associated with central fat distribution. Also, 5α-reductase activity which is associated with adipose tissue, increased with indexes of insulin resistance [39]. On the other hand, visceral adiposity [40], which is intensified by the aging process [41], has been announced to be a primary trigger for most of the pathways involved in the MetS especially for insulin resistance [42]. It seems that changes in hormone levels in individuals, which are influenced by the aging process and trigger metabolic changes in humans, can largely explain some of these differences. In Chedraui et al.’s study [43], an increase in IL-6 and a decrease in urokinase-type plasminogen activator levels in postmenopausal women have been introduced as the reasons for the effects of these hormonal changes on the occurrence of the MetS. Also, in relation to the more initiating effects of hypertension in the higher age group in women, the effect of endocrine system activity regress, which reflects changes in hormone levels, has been noted [44]. Here, the key issue is the combination of the triggers and their comorbidity, which partly justifies the rising trend of the MetS due to the cumulative effects of the components over time and with age. In other words, approximately, no studies have addressed the role of an isolated component in the create conditions for subsequent occurrence of the MetS. This may be an indication that metabolic components tend to co-occur, suggesting a close association and shared roots. Many researchers believe that the four components of the MetS are overlapping in their pathogenic activities, and this occurs through similar metabolic pathways [45]. Therefore, it is advisable to check the other components carefully and periodically for people who usually have one of the components. Therefore, it should be said that there are two important issues in relation to the triggers in different studies; one is its combination, and the other is its demographic variability, which justifies both the rising trend with age and gender difference based on hormonal differences and interplays. In general, it seems that the role of overweight/obesity and dyslipidemia components in the age group below the age of 50 and the role of hypertension in the age group over the age of 50 were be explained by hormonal changes. Of the various available studies, none has mentioned to hyperglycemia as a trigger. Dyslipidemia is said to play a mediating role in the free fatty acid (FFA) damage to the islet function process (which causes hypertension and hyperglycemia) [19, 46]. So, at first glance, the combination of dyslipidemia with hypertension may seem justifiable in the development of the MetS. On the other hand, overweight/obesity introduced also as an important risk factor for dyslipidemia and hypertension and hyperglycemia as major components of the MetS, especially in younger people [47, 48]. Even elsewhere, it, especially abdominal obesity, has been independently introduced as the most important cause of the MetS [49]. Advocators of this hypothesis that point to the higher value of this component than other components at the onset of the MetS, using the same argument, namely temporal priority of the overweight/obesity component over others and suggests that this component is, in fact, an important risk factor for the subsequent occurrence of other components [47]. However, the causal link between dyslipidemia and overweight/obesity remains a controversial issue [16]. Also, in this study, TP values from no component to MetS were higher in all Markovian studies in men than in women; With the exception of one study in which the transition from no component to overweight/obesity and low HDL states and the TP from overweight/obesity to the MetS after the age of 50 was higher in women. The latter finding, namely the higher TP from no component to overweight/obesity and low HDL states, as well as the possibility of transition from overweight/obesity to the MetS in women, is largely attributable to hormonal differences between men and women after 50 years old. However, with age increasing, the gap between men and women gradually decreased except for the recent exception noted previously. Also, in the Markov model, the probabilities of reverse transitions from each of the states to the no component state were lower in men than in women; However, these probabilities decreased significantly with age. These findings primarily indicate that men at higher risk (in terms of components) are more likely to develop the MetS than women, and are therefore more resistant to return to the normal state. As mentioned earlier, the central triggers in this study included the components of dyslipidemia, overweight/obesity and hypertension. On the other hand, although its mechanism is not yet known, dyslipidemia is claimed to cause overweight/obesity and hypertension in men more than women [19, 50]. The some of the higher developmental trend of the MetS in men may be due to this issue. Interestingly, most studies [51–55] have been reported a higher prevalence of the MetS in women than men but in all studies (Markovian studies) on the natural history of the MetS, it has been said that men have a higher rate of progression to the MetS than women. Various studies have shown that weight gain, hypertension, and hyperglycemia are important risk factors for dyslipidemia [56, 57]. On the other hand, the prevalence of all these factors has been reported higher in women than men [58]. Thus, the speed of the MetS development is expected to be higher (as the prevalence of the MetS) in women than men, but despite the evidence available for faster growth of the MetS in women than men, the accumulation of evidence is in favor of the higher speed of the MetS development in men than women. The prevalence and clustering speed of the components appear to be higher in men than in women [59], and this may be partly responsible for the faster occurrence of the disorder in men. One of the individual studies used in this review [19] has also shown that after hyperglycemia, men are more likely to develop hypertension and, as a result, different forms of multi-component, which is in line with the recent justification. But it may be possible to attribute the higher rate of the MetS development in men than women to the important problem of androgen deficiency in men in the best way [60]. Men with the MetS relative to women are said to be at higher risk for androgen deficiency and Late-onset hypogonadism or Testosterone Deficiency Syndrome. The pathophysiology of these disorders is multifactorial and contains defective inflammatory, enzymatic, and endocrine mechanisms that provide the basis for further development of the MetS in patients so that by treating these mechanisms, the parameters of the MetS are improved [60–63]. Overall, the most common transitions (with a high TP) with the Markovian approach were primarily due to the transition from dyslipidemia to no component, and second, the transition from all-states to 2-component. Regarding the high transition from dyslipidemia to no component, it should probably be said that, in the first place, much of this, is due to the success of dyslipidemia treatment, which response better to treatment compared to other components (whether medications or lifestyle changes). However, in the articles used in this review, the treatment status is unclear, so this argument is only a speculation. Without any treatment, in the long-term, it seems that natural changes in lifestyle and related events can lead to lower lipid profile and normalization to other components of the MetS. Secondly, it must be said that in this transition, there may be several biological and physiological mechanisms, such as the effect of thyroid hormones [64, 65], female hormone interactions in women [66] and the effect of preventive actions on other components on lipid profile [67, 68] that due to the interdependence of the components and the pathophysiological mechanisms between them or unknown self-therapies, they play a role that is not mentioned in the articles used.

The high transition from all states to the 2-component state can also be mainly due to the natural development of isolated components toward complete the MetS establishment that discussed earlier. However, in our study, also the TP from any of the 2-component states to the MetS, which is a continuation of the natural history of the MetS, was also high. Also, the least TP occurred from hypertension, dyslipidemia, and hyperglycemia to each of the model states except for 2-component and MetS states and from the MetS state to all states except hypertension and 2-component states that all of them, in other words, indicate a high rate of progress toward the establishment of the MetS. Regarding the reverse transition from MetS to 2-component state in this process, it is possible to point to a similar mechanism as that described for the reverse transition to no component state. However, the dynamics mentioned in the natural process of the MetS development suggest that this process is influenced by various factors, that many of which are still unknown, and their discovery requires detailed studies of the natural history of the MetS in the other general populations.

Regarding the most common combinations with the Markovian approach, the combination of dyslipidemia with overweight/obesity should be noted and the combination of hyperglycemia and overweight/obesity with the non-Markovian approach. The commonality of both approaches is the overweight/obesity component. Combining dyslipidemia with overweight/obesity is a common combination, because obesity increase triglyceride and LDL, and decrease HDL as components of dyslipidemia, as well as elevated blood glucose and insulin levels through various pathways such as hepatic overproduction of VLDL and decreased circulating TG lipolysis [69]. However, one should not overlook the role of age; however, with Markov’s approach, the probability of combining dyslipidemia with overweight/obesity was higher in the under-50 age group, and in the over-50 age group the probability of combining dyslipidemia with hypertension was higher [19]. Regarding the non-Markovian approach, it should be said that this approach is mainly due to the use of predictive and regression-based statistical models and, of course, it has a serious difference with the Markovian models approach that looks at longitudinal and more realistic to evolution of a phenomenon over time and also the possibility of various errors, there is no evidence-based judgment regarding the combination of hyperglycemia and overweight/obesity. However, similar to the argument used in the Markovian approach, the role of the overweight/obesity component for providing insulin resistance and hyperglycemia in relation to other components may be mentioned that provides the higher linkage between the two components and is discussed elsewhere [70]. Finally, with the Markovian approach, the most important components, which in the simulation process, increasingly were moving toward a full establishment of the MetS, were 2-component states and hyperglycemia and, the most important predictor of the MetS occurrence with non-Markovian was overweight/obesity. The importance of 2-component states in the development and establishment of the MetS in individuals reflects the cumulative effects of the components together. Although it is shown that in isolated component states, the probability of developing the MetS over the next 10 years is higher than in no component states that of course, with the Markovian approach, as shown, hyperglycemia is progressively more likely to lead to the MetS among the isolated components. Also, it is shown that in 2-component states, the highest probability of establishing the MetS appears in the first 5 years [4, 71]. Regarding hyperglycemia, also it should be said the effect of insulin resistance on the subsequent development of the MetS is a well-defined pathologic process and is often associated with the occurrence of other components of the MetS [49, 72]. Regarding non-Markovian approaches, as stated earlier, regression models have been used for prediction in most of the studies and due to the different nature of the statistical analyses and approaches to prediction in this model compared to the Markovian model that using the simulation process, no evidence-based judgment can be made. In spite of this, new evidence is in favor of the parallel development of the obesity epidemic with the MetS, especially in children and adolescents, and obesity is routinely used and evaluated in studies with non-Markovian approaches in these areas. On the other hand, the role of obesity in the pathogenesis of insulin resistance has been established as a key factor in the development of the MetS, and it is obvious that the overweight/obesity in a well-designed regression model provides a good estimate of the MetS.

The strength of our study is that it is for the first time that the process of the natural history of the MetS, especially summarize of studies conducted with the Markov model, in the form of a comprehensive review is discussed. On the other hand, the lack of primary studies in this field which reduced the possibility of pros and cons discussions with available evidences, also, difficultness of understanding of inferences made from Markov model processes for non-specialists were of the most important limitations of this study which of course, it was tried to help to better understand the concepts as much as possible by simplifying the explanations and interpretations.

## Conclusion

In our study, the role of Markov modeling as well as importance of some components, such as dyslipidemia and hypertension in development of the MetS compared with others was emphasized. From this perspective, the most important trigger components, combinations along with their predictive role for getting the MetS in the future were dyslipidemia and hypertension and combination of dyslipidemia with obesity or hyperglycemia. It seems a forward-looking perspective on the interaction between components and importance of each component in predicting the MetS that can be used in preventive and intervention discussions in high-risk individuals for drug interventions or lifestyle-based changes is required in the study process of MetS to better understanding of disease establishment conditions.

## Supplementary Information

**Additional file 1 Table 7** TP of no component state to other states in studies. **Table 8** TP of overweight or obesity state to other states in studies. **Table 9** TP of hypertension state to other states in studies. **Table 10** TP of dyslipidemia state to other states in studies. **Table 11** TP of hyperglycemia state to other states in studies. **Table 12** TP of “Overweight / obesity & hypertension” state to other states in studies. **Table 13** TP of “Overweight / obesity & hyperglycemia” state to other states in studies. **Table 14** TP of “Overweight / obesity & hyperglycemia” state to other states in studies. **Table 15** TP of “hypertension & dyslipidemia” state to other states in studies. **Table 16** TP of “hypertension & hyperglycemia” state to other states in studies. **Table 17** TP of “dyslipidemia & hyperglycemia” state to other states in studies. **Table 18** TP of “MetS” state to other states in studies.

## Data Availability

The datasets used and/or analyzed during the current study are available from the corresponding author on reasonable request.

## Abbreviations

MetS: Metabolic syndrome
PRISMA: Preferred Reporting Items for Systematic Reviews and Meta-Analyses
HDL: High-density lipoprotein
CVD: Cardiovascular disease
PICO/PECO: Population, Intervention/Exposure, Comparison, Outcome
TP: Transition probabilities
CDS: Chinese Diabetes Society
NCEP: National Cholesterol Educational Program
IDF: International diabetes federation
AHA/NHLBI: American Heart Association and the National Heart, Lung, and Blood Institute
WHR: Waist-to-Hip Ratio
