# Supplementary material for: Dynamic behavior of metabolic syndrome progression: a comprehensive systematic review on recent discoveries
Source: BMC Endocr Disord. 2021 Mar 22;21:54. doi: 10.1186/s12902-021-00716-7 (PMC7986266; doi:10.1186/s12902-021-00716-7)
Supplement: Supplementary file 1 — Additional file 1 Table 7 TP of no component state to other states in studies. Table 8 TP of overweight or obesity state to other states in studies. Table 9 TP of hypertension state to other states in studies. Table 10 TP of dyslipidemia state to other states in studies. Table 11 TP of hyperglycemia state to other states in studies. Table 12 TP of “Overweight / obesity & hypertension” state to other states in studies. Table 13 TP of “Overweight / obesity & hyperglycemia” state to other states in studies. Table 14 TP of “Overweight / obesity & hyperglycemia” state to other states in studies. Table 15 TP of “hypertension & dyslipidemia” state to other states in studies. Table 16 TP of “hypertension & hyperglycemia” state to other states in studies. Table 17 TP of “dyslipidemia & hyperglycemia” state to other states in studies. Table 18 TP of “MetS” state to other states in studies. [file 12902_2021_716_MOESM1_ESM.docx]

Supplement:

Table 7) TP of no component state to other states in studies

|  | | | No component | overweight/ obesity | hypertension | dyslipidemia | hyperglycemia | 2-component | ≥3 component (MS) |
| --- | --- | --- | --- | --- | --- | --- | --- | --- | --- |
| No component | Xiaoxian Jia | for men in the 18–49-year group | *66.44* | 10.15 | 4.19 | 10.78 | .67 | 1.08 | 1.28 |
|  |  | for women in the 18–49-year group | *78.21* | 7.15 | 2.44 | 8.45 | .60 | .42 | .63 |
|  |  | for men in the ≥ 50-year group | *60.42* | 5.09 | 12.11 | 11.44 | 2.26 | 1.19 | 1.54 |
|  |  | for women in the ≥ 50-year group | *64.86* | 4.01 | 8.17 | 11.30 | 2.04 | 1.35 | 1.5 |
|  | Xiaoxiao Chen | for men in the 18–40-year group | *72.07* | 9.48 | 4.55 | 7.68 | .58 | 4.55 | 1.09 |
|  |  | for women in the 18–40-year group | *86.38* | 3.71 | 2.15 | 3.76 | .80 | 2.30 | .9 |
|  |  | for men in the 40–49-year group | *68.15* | 8.30 | 4.26 | 10.54 | 1.57 | 5.72 | 1.46 |
|  |  | for women in the 40–49-year group | *82.05* | 5.45 | 3.7 | 4.61 | .84 | 2.65 | .7 |
|  |  | for men in the 50–59-year group | *62.71* | 9.9 | 5.94 | 7.92 | 3.96 | 7.59 | 1.98 |
|  |  | For women in the 50-59-year group | 69.18 | 5.41 | 5.95 | 9.73 | 0.54 | 7.57 | 1.62 |
|  |  | for men in the ≥ 60 year group | 65.98 | 6.19 | 11.34 | 5.15 | 2.06 | 6.19 | 3.09 |
|  |  | for women in the ≥60 year group | 58.34 | 8.33 | 8.33 | 13.33 | 1.67 | 8.33 | 1.67 |
|  | Xiao Tang | for men in the 20- to 40-year-old age group | *76.40* | 2.86 | 6.72 | 1.29 | 7.30 | 4.72 | .71 |
|  |  | for men in the 40–60-year-old age group | *67.63* | 6.81 | 5.79 | 2.04 | 11.24 | 5.46 | 1.03 |
|  |  | for women in the 20- to 40-year-old age group | *87.04* | 1.70 | 3.85 | .81 | 3.70 | 2.15 | .75 |
|  |  | for women in the 40- to 60-year-old age group | *83.78* | 3.39 | 3.85 | 1.64 | 4.85 | 1.98 | .51 |
|  | Lee-Ching Hwang | men | *92.89* | .91 | 1.99 | .91 | .2 | 1.73 | .45 |
|  |  | women | *92.82* | 1.67 | .41 | 1.525 | .16 | 1.49 | .41 |

Table 8) TP of overweight or obesity state to other states in studies

|  | | | No component | overweight/ obesity | hypertension | dyslipidemia | hyperglycemia | 2-component | ≥3 component (MS) |
| --- | --- | --- | --- | --- | --- | --- | --- | --- | --- |
| overweight  or obesity | Xiaoxian Jia | for men in the 18–49 year group | 7.14 | *47.19* | 1.12 | 2.7 | .16 | 5.5 | 8.63 |
|  |  | for women in the 18–49 year group | 18.51 | *52.01* | .95 | 4.32 | .63 | 3.41 | 3.07 |
|  |  | for men in the ≥ 50 year group | 8.97 | *40.96* | 1.65 | 1.62 | 0 | 6.18 | 9.70 |
|  |  | for women in the ≥ 50 year group | 12.08 | *40.30* | .78 | 5.38 | 2.46 | 5.343 | 6.93 |
|  |  | for men in the 18–40 year group | 13.51 | *52.25* | 1.03 | 3.35 | .51 | 24.07 | 5.28 |
|  |  | for women in the 18–40 year group | 37.81 | *44.27* | .5 | 4.98 | .5 | 9.45 | 2.49 |
|  |  | for men in the 40–49 year group | 13.17 | *52.23* | 0 | 3.13 | .22 | 25.45 | 5.8 |
|  |  | for women in the 40–49 year group | 27.5 | *54.16* | 1.25 | 4.17 | .42 | 11.25 | 1.25 |
|  |  | for men in the 50–59 year group | 18.62 | *46.28* | 2.66 | 4.79 | .53 | 20.21 | 6.91 |
|  |  | For women in the 50-59 year group | 19.61 | 41.18 | 1.96 | 3.92 | 1.96 | 19.61 | 11.76 |
|  |  | for men in the ≥ 60 year group | 20.63 | 49.21 | 0 | 1.59 | 0 | 26.98 | 1.59 |
|  |  | for women in the ≥60 year group | 25.81 | 51.60 | 3.23 | 0 | 0 | 16.13 | 3.23 |
|  | Xiao Tang | for men in the 20- to 40-year-old age group | 9.25 | *55.25* | .25 | 1 | .5 | 28.75 | 5 |
|  |  | for men in the 40–60-year-old age group | 6.94 | *55.57* | .83 | .83 | .83 | 27.78 | 7.22 |
|  |  | for women in the 20- to 40-year-old age group | 25.78 | *54.95* | 1.3 | 2.08 | .26 | 12.24 | 3.39 |
|  |  | for women in the 40- to 60-year-old age group | 13.5 | *62.26* | 0 | 1.93 | 0 | 18.73 | 3.58 |
|  | Lee-Ching Hwang | men | 2.16 | *86.77* | .35 | .525 | .01 | 6.76 | 2.93 |
|  |  | women | 2.22 | *85.42* | .01 | .135 | .01 | 6.39 | 5.70 |

Table 9) TP of hypertension state to other states in studies

|  | | | No component | overweight/ obesity | hypertension | dyslipidemia | hyperglycemia | 2-component | ≥3 component (MS) |
| --- | --- | --- | --- | --- | --- | --- | --- | --- | --- |
| hypertension | Xiaoxian Jia | for men in the 18–49 year group | 0 | 0 | *63.81* | 0 | 0 | 4.66 | 8.21 |
|  |  | for women in the 18–49 year group | 0 | 0 | *74.82* | 0 | 0 | 3.636 | 3.37 |
|  |  | for men in the ≥ 50 year group | 0 | 0 | *62.64* | 0 | 0 | 4.96 | 7.56 |
|  |  | for women in the ≥ 50 year group | 0 | 0 | *56.31* | 0 | 0 | 6.25 | 6.17 |
|  | Xiaoxiao Chen | for men in the 18–40 year group | 0 | 0 | *61.21* | 0 | 0 | 26.67 | 12.12 |
|  |  | for women in the 18–40 year group | 0 | 0 | *83.82* | 0 | 0 | 13.24 | 2.94 |
|  |  | for men in the 40–49 year group | 0 | 0 | *64.71* | 0 | 0 | 27.01 | 8.29 |
|  |  | for women in the 40–49 year group | 0 | 0 | *86.80* | 0 | 0 | 11.46 | 1.74 |
|  |  | for men in the 50–59 year group | 0 | 0 | *66.40* | 0 | 0 | 29.25 | 4.35 |
|  |  | For women in the 50-59 year group | 0 | 0 | 65.62 | 0 | 0 | 31.25 | 3.13 |
|  |  | for men in the ≥ 60 year group | 0 | 0 | 73.81 | 0 | 0 | 25.40 | 0.79 |
|  |  | for women in the ≥60 year group | 0 | 0 | 53.45 | 0 | 0 | 32.76 | 13.79 |
|  | Xiao Tang | for men in the 20- to 40-year-old age group | 16.95 | 8.48 | *49.16* | 1.69 | 1.69 | 16.95 | 5.08 |
|  |  | for men in the 40–60-year-old age group | 13.11 | 4.84 | *48.99* | 2.42 | 2.42 | 21.77 | 6.45 |
|  |  | for women in the 20- to 40-year-old age group | 24.26 | 7.92 | *52.97* | 3.96 | 0 | 8.91 | 1.98 |
|  |  | for women in the 40- to 60-year-old age group | 13.69 | 1.06 | *61.43* | 1.59 | 2.12 | 17.46 | 2.65 |
|  | Lee-Ching Hwang | men | 6.10 | .32 | *85.66* | .8 | .64 | 3.38 | 2.31 |
|  |  | women | 4.23 | .01 | *83.91* | 1.615 | .01 | 6.19 | 2.44 |

Table 10) TP of dyslipidemia state to other states in studies

|  | | | No component | overweight/ obesity | hypertension | dyslipidemia | hyperglycemia | 2-component | ≥3 component (MS) |
| --- | --- | --- | --- | --- | --- | --- | --- | --- | --- |
| dyslipidemia | Xiaoxian Jia | for men in the 18–49-year group | 23.04 | 3.14 | 2.85 | *48.02* | 0 | 2.88 | 5.61 |
|  |  | for women in the 18–49-year group | 39.90 | 1.37 | 2.04 | *46.36* | 0 | 1.43 | 1.72 |
|  |  | for men in the ≥ 50 year group | 23.89 | 2.32 | 3.17 | *41.93* | 0 | 3.34 | 8.63 |
|  |  | for women in the ≥ 50 year group | 23.89 | 1.96 | 1.92 | *52.69* | 2.22 | 1.95 | 5.61 |
|  | Xiaoxiao Chen | for men in the 18–40 year group | 23.21 | 3.27 | 1.79 | *42.86* | .3 | 22.32 | 6.25 |
|  |  | for women in the 18–40 year group | 47.57 | 5.83 | 2.91 | *25.24* | .97 | 10.68 | 6.8 |
|  |  | for men in the 40–49 year group | 24.82 | 4.61 | 3.19 | *37.59* | 1.06 | 23.05 | 5.67 |
|  |  | for women in the 40–49 year group | 38.25 | 3.48 | 3.48 | *42.61* | 0 | 9.57 | 2.61 |
|  |  | for men in the 50–59 year group | 22.97 | 1.35 | 1.35 | *36.49* | 2.7 | 29.73 | 5.41 |
|  |  | For women in the 50-59 year group | 42.86 | 7.14 | 0 | 21.43 | 0 | 21.43 | 7.14 |
|  |  | for men in the ≥ 60 year group | 8.00 | 4.00 | 4.00 | 56.00 | 4.00 | 24.00 | 0 |
|  |  | for women in the ≥60 year group | 19.05 | 0 | 9.52 | 38.10 | 4.76 | 19.05 | 9.52 |
|  | Xiao Tang | for men in the 20- to 40-year-old age group | 21.28 | 5.16 | 2.58 | *41.94* | 1.29 | 20.65 | 7.10 |
|  |  | for men in the 40–60-year-old age group | 25.37 | 3.42 | 3.41 | *43.41* | 1.46 | 15.61 | 7.32 |
|  |  | for women in the 20- to 40-year-old age group | 55.11 | 4.89 | 4.89 | *26.67* | 1.33 | 6.67 | .44 |
|  |  | for women in the 40- to 60-year-old age group | 40.96 | 3.72 | 1.6 | *37.23* | 2.13 | 13.3 | 1.06 |
|  | Lee-Ching Hwang | men | 8.77 | .13 | 1.53 | *41.14* | .13 | 4.955 | 2.125 |
|  |  | women | 7.91 | .965 | .585 | *42.78* | .01 | 3.58 | 1.58 |

Table 11) TP of hyperglycemia state to other states in studies

|  | | | No component | overweight/ obesity | hypertension | dyslipidemia | hyperglycemia | 2-component | ≥3 component (MS) |
| --- | --- | --- | --- | --- | --- | --- | --- | --- | --- |
| hyperglycemia | Xiaoxian Jia | for men in the 18–49 year group | 0 | 0 | 0 | 0 | *45.66* | 7.095 | 11.76 |
|  |  | for women in the 18–49 year group | 0 | 0 | 0 | 0 | *67.46* | 4.72 | 4.22 |
|  |  | for men in the ≥ 50 year group | 0 | 0 | 0 | 0 | *47.32* | 6.38 | 14.41 |
|  |  | for women in the ≥ 50 year group | 0 | 0 | 0 | 0 | *55.98* | 5.356 | 11.87 |
|  | Xiaoxiao Chen | for men in the 18–40 year group | 0 | 0 | 0 | 0 | *48.57* | 31.43 | 20 |
|  |  | for women in the 18–40 year group | 0 | 0 | 0 | 0 | *88.64* | 9.09 | 2.27 |
|  |  | for men in the 40–49 year group | 0 | 0 | 0 | 0 | *56.90* | 31.03 | 12.07 |
|  |  | for women in the 40–49 year group | 0 | 0 | 0 | 0 | *73.81* | 19.05 | 7.14 |
|  |  | for men in the 50–59 year group | 0 | 0 | 0 | 0 | *53.57* | 30.36 | 16.07 |
|  |  | For women in the 50-59 year group | 0 | 0 | 0 | 0 | 71.43 | 28.57 | 0 |
|  |  | for men in the ≥ 60 year group | 0 | 0 | 0 | 0 | 50.00 | 43.75 | 6.25 |
|  |  | for women in the ≥60 year group | 0 | 0 | 0 | 0 | 38.46 | 30.77 | 30.77 |
|  | Xiao Tang | for men in the 20- to 40-year-old age group | 22.41 | 3.45 | 6.9 | 6.9 | *43.10* | 6.9 | 10.34 |
|  |  | for men in the 40–60-year-old age group | 12.16 | 1.35 | 1.35 | 1.35 | *52.71* | 18.92 | 12.16 |
|  |  | for women in the 20- to 40-year-old age group | 23.28 | 0 | 1.72 | 3.45 | *57.76* | 12.07 | 1.72 |
|  |  | for women in the 40- to 60-year-old age group | 20.93 | 1.16 | 3.49 | 2.33 | *51.16* | 17.44 | 3.49 |
|  | Lee-Ching Hwang | men | 11.29 | .9 | 1.84 | .92 | *79.41* | 3.84 | .9 |
|  |  | women | 6.7 | 5.19 | .59 | 1.515 | *80.81* | 3.10 | .59 |

Table 12) TP of “Overweight / obesity & hypertension” state to other states in studies

|  | | | No component | overweight/ obesity | hypertension | dyslipidemia | hyperglycemia | 2-component | ≥3 component (MS) |
| --- | --- | --- | --- | --- | --- | --- | --- | --- | --- |
| Overweight  or obesity  and hypertension | Xiaoxian Jia | for men in the 18–49 year group | 0 | 0 | 7.24 | 0 | 0 | 11.475 | 23.91 |
|  |  | for women in the 18–49 year group | 0 | 0 | 17.75 | 0 | 0 | 10.98 | 16.32 |
|  |  | for men in the ≥ 50 year group | 0 | 0 | 9.28 | 0 | 0 | 10.54 | 27.49 |
|  |  | for women in the ≥ 50 year group | 0 | 0 | 6.47 | 0 | 0 | 12.158 | 20.58 |
|  | Xiaoxiao Chen | for men in the 18–40 year group | 3.33 | 9.07 | 9.44 | 2.5 | .83 | 54.17 | 20.65 |
|  |  | for women in the 18–40 year group | 7.69 | 6.51 | 20.71 | 2.37 | 3.55 | 46.74 | 12.43 |
|  |  | for men in the 40–49 year group | 1.28 | 4.81 | 8.49 | 3.13 | 1.44 | 57.69 | 23.16 |
|  |  | for women in the 40–49 year group | 3.16 | 3.95 | 21.34 | 3.16 | 3.95 | 49.02 | 15.42 |
|  |  | for men in the 50–59 year group | .53 | 2.79 | 7.85 | 1.6 | 1.33 | 60.24 | 25.66 |
|  |  | For women in the 50-59 year group | 0.56 | 3.39 | 20.90 | 1.69 | 0.56 | 53.13 | 19.77 |
|  |  | for men in the ≥ 60 year group | 1.58 | 2.77 | 11.07 | 0.79 | 1.98 | 55.72 | 26.09 |
|  |  | for women in the ≥60 year group | 1.09 | 0.54 | 10.87 | 0 | 2.72 | 56.52 | 28.26 |
|  | Xiao Tang | for men in the 20- to 40-year-old age group | 5.98 | 21.56 | 2 | 4.39 | .8 | 50.90 | 14.37 |
|  |  | for men in the 40–60-year-old age group | 2.81 | 13.04 | 3.07 | 4.22 | 2.05 | 51.79 | 23.02 |
|  |  | for women in the 20- to 40-year-old age group | 24.68 | 20.85 | 5.11 | 3.83 | 2.55 | 34.47 | 8.51 |
|  |  | for women in the 40- to 60-year-old age group | 8.65 | 14.05 | 11.90 | 4.86 | 3.78 | 45.41 | 11.35 |
|  | Lee-Ching Hwang | men | 2.27 | 1.29 | .81 | .93 | .58 | 86.33 | 6.87 |
|  |  | women | 4.82 | 1.55 | .85 | .95 | .01 | 86.67 | 4.21 |

Table 13) TP of “Overweight / obesity & hyperglycemia” state to other states in studies

|  | | | No component | overweight/ obesity | hypertension | dyslipidemia | hyperglycemia | 2-component | ≥3 component (MS) |
| --- | --- | --- | --- | --- | --- | --- | --- | --- | --- |
| Overweight  or obesity  and hyperglycemia | Xiaoxian Jia | for men in the 18–49 year group | 0 | 0 | 0 | 0 | 7.74 | 10.84 | 27.20 |
|  |  | for women in the 18–49 year group | 0 | 0 | 0 | 0 | 17.37 | 10.20 | 21.42 |
|  |  | for men in the ≥ 50 year group | 0 | 0 | 0 | 0 | 7.03 | 10.245 | 31.49 |
|  |  | for women in the ≥ 50 year group | 0 | 0 | 0 | 0 | 10.61 | 10.368 | 27.18 |
|  | Xiaoxiao Chen | for men in the 18–40 year group | 3.33 | 9.07 | 9.44 | 2.5 | .83 | 54.17 | 20.65 |
|  |  | for women in the 18–40 year group | 7.69 | 6.51 | 20.71 | 2.37 | 3.55 | 46.74 | 12.43 |
|  |  | for men in the 40–49 year group | 1.28 | 4.81 | 8.49 | 3.13 | 1.44 | 57.69 | 23.16 |
|  |  | for women in the 40–49 year group | 3.16 | 3.95 | 21.34 | 3.16 | 3.95 | 49.02 | 15.42 |
|  |  | for men in the 50–59 year group | .53 | 2.79 | 7.85 | 1.6 | 1.33 | 60.24 | 25.66 |
|  |  | For women in the 50-59 year group | 0.56 | 3.39 | 20.90 | 1.69 | 0.56 | 53.13 | 19.77 |
|  |  | for men in the ≥ 60 year group | 1.58 | 2.77 | 11.07 | 0.79 | 1.98 | 55.72 | 26.09 |
|  |  | for women in the ≥60 year group | 1.09 | 0.54 | 10.87 | 0 | 2.72 | 56.52 | 28.26 |
|  | Xiao Tang | for men in the 20- to 40-year-old age group | 5.98 | 21.56 | 2 | 4.39 | .8 | 50.90 | 14.37 |
|  |  | for men in the 40–60-year-old age group | 2.81 | 13.04 | 3.07 | 4.22 | 2.05 | 51.79 | 23.02 |
|  |  | for women in the 20- to 40-year-old age group | 24.68 | 20.85 | 5.11 | 3.83 | 2.55 | 34.47 | 8.51 |
|  |  | for women in the 40- to 60-year-old age group | 8.65 | 14.05 | 11.90 | 4.86 | 3.78 | 45.41 | 11.35 |
|  | Lee-Ching Hwang | men | 2.27 | 1.29 | .81 | .93 | .58 | 86.33 | 6.87 |
|  |  | women | 4.82 | 1.55 | .85 | .95 | .01 | 86.67 | 4.21 |

Table 14) TP of “Overweight / obesity & hyperglycemia” state to other states in studies

|  | | | No component | overweight/ obesity | hypertension | dyslipidemia | hyperglycemia | 2-component | ≥3 component (MS) |
| --- | --- | --- | --- | --- | --- | --- | --- | --- | --- |
| Overweight  or obesity  and dyslipidemia | Xiaoxian Jia | for men in the 18–49 year group | 3.14 | 15.08 | 0 | 4.86 | 0 | 10.34 | 14.83 |
|  |  | for women in the 18–49 year group | 9.33 | 21.16 | 0 | 12.99 | .34 | 8.098 | 7.57 |
|  |  | for men in the ≥ 50 year group | 0 | 0 | 21.42 | 0 | 0 | 10.393 | 16.22 |
|  |  | for women in the ≥ 50 year group | 6.22 | 13.99 | 0 | 6.42 | 0 | 9.351 | 17.27 |
|  | Xiaoxiao Chen | for men in the 18–40 year group | 3.33 | 9.07 | 9.44 | 2.5 | .83 | 54.17 | 20.65 |
|  |  | for women in the 18–40 year group | 7.69 | 6.51 | 20.71 | 2.37 | 3.55 | 46.74 | 12.43 |
|  |  | for men in the 40–49 year group | 1.28 | 4.81 | 8.49 | 3.13 | 1.44 | 57.69 | 23.16 |
|  |  | for women in the 40–49 year group | 3.16 | 3.95 | 21.34 | 3.16 | 3.95 | 49.02 | 15.42 |
|  |  | for men in the 50–59 year group | .53 | 2.79 | 7.85 | 1.6 | 1.33 | 60.24 | 25.66 |
|  |  | For women in the 50-59 year group | 0.56 | 3.39 | 20.90 | 1.69 | 0.56 | 53.13 | 19.77 |
|  |  | for men in the ≥ 60 year group | 1.58 | 2.77 | 11.07 | 0.79 | 1.98 | 55.72 | 26.09 |
|  |  | for women in the ≥60 year group | 1.09 | 0.54 | 10.87 | 0 | 2.72 | 56.52 | 28.26 |
|  | Xiao Tang | for men in the 20- to 40-year-old age group | 5.98 | 21.56 | 2 | 4.39 | .8 | 50.90 | 14.37 |
|  |  | for men in the 40–60-year-old age group | 2.81 | 13.04 | 3.07 | 4.22 | 2.05 | 51.79 | 23.02 |
|  |  | for women in the 20- to 40-year-old age group | 24.68 | 20.85 | 5.11 | 3.83 | 2.55 | 34.47 | 8.51 |
|  |  | for women in the 40- to 60-year-old age group | 8.65 | 14.05 | 11.90 | 4.86 | 3.78 | 45.41 | 11.35 |
|  | Lee-Ching Hwang | men | 2.27 | 1.29 | .81 | .93 | .58 | 86.33 | 6.87 |
|  |  | women | 4.82 | 1.55 | .85 | .95 | .01 | 86.67 | 4.21 |

Table 15) TP of “hypertension & dyslipidemia” state to other states in studies

|  | | | No component | overweight/ obesity | hypertension | dyslipidemia | hyperglycemia | 2-component | ≥3 component (MS) |
| --- | --- | --- | --- | --- | --- | --- | --- | --- | --- |
| Hypertension  and dyslipidemia | Xiaoxian Jia | for men in the 18–49 year group | 0 | 0 | 26.15 | 0 | 0 | 10 | 13.82 |
|  |  | for women in the 18–49 year group | 0 | 0 | 23.20 | 0 | 0 | 10.99 | 10.87 |
|  |  | for men in the ≥ 50 year group | 0 | 0 | 21.42 | 0 | 0 | 10.393 | 16.22 |
|  |  | for women in the ≥ 50 year group | 0 | 0 | 20.05 | 0 | 0 | 10.815 | 15.07 |
|  | Xiaoxiao Chen | for men in the 18–40 year group | 3.33 | 9.07 | 9.44 | 2.5 | .83 | 54.17 | 20.65 |
|  |  | for women in the 18–40 year group | 7.69 | 6.51 | 20.71 | 2.37 | 3.55 | 46.74 | 12.43 |
|  |  | for men in the 40–49 year group | 1.28 | 4.81 | 8.49 | 3.13 | 1.44 | 57.69 | 23.16 |
|  |  | for women in the 40–49 year group | 3.16 | 3.95 | 21.34 | 3.16 | 3.95 | 49.02 | 15.42 |
|  |  | for men in the 50–59 year group | .53 | 2.79 | 7.85 | 1.6 | 1.33 | 60.24 | 25.66 |
|  |  | For women in the 50-59 year group | 0.56 | 3.39 | 20.90 | 1.69 | 0.56 | 53.13 | 19.77 |
|  |  | for men in the ≥ 60 year group | 1.58 | 2.77 | 11.07 | 0.79 | 1.98 | 55.72 | 26.09 |
|  |  | for women in the ≥60 year group | 1.09 | 0.54 | 10.87 | 0 | 2.72 | 56.52 | 28.26 |
|  | Xiao Tang | for men in the 20- to 40-year-old age group | 5.98 | 21.56 | 2 | 4.39 | .8 | 50.90 | 14.37 |
|  |  | for men in the 40–60-year-old age group | 2.81 | 13.04 | 3.07 | 4.22 | 2.05 | 51.79 | 23.02 |
|  |  | for women in the 20- to 40-year-old age group | 24.68 | 20.85 | 5.11 | 3.83 | 2.55 | 34.47 | 8.51 |
|  |  | for women in the 40- to 60-year-old age group | 8.65 | 14.05 | 11.90 | 4.86 | 3.78 | 45.41 | 11.35 |
|  | Lee-Ching Hwang | men | 2.27 | 1.29 | .81 | .93 | .58 | 86.33 | 6.87 |
|  |  | women | 4.82 | 1.55 | .85 | .95 | .01 | 86.67 | 4.21 |

Table 16) TP of “hypertension & hyperglycemia” state to other states in studies

|  | | | No component | overweight/ obesity | hypertension | dyslipidemia | hyperglycemia | 2-component | ≥3 component (MS) |
| --- | --- | --- | --- | --- | --- | --- | --- | --- | --- |
| Hypertension  and hyperglycemia | Xiaoxian Jia | for men in the 18–49 year group | 0 | 0 | 0 | 0 | 0 | 13.2 | 20.80 |
|  |  | for women in the 18–49 year group | 0 | 0 | 0 | 0 | 0 | 13.24 | 20.56 |
|  |  | for men in the ≥ 50 year group | 0 | 0 | 0 | 0 | 0 | 12.245 | 26.53 |
|  |  | for women in the ≥ 50 year group | 0 | 0 | 0 | 0 | 0 | 12.50 | 24.99 |
|  | Xiaoxiao Chen | for men in the 18–40 year group | 3.33 | 9.07 | 9.44 | 2.5 | .83 | 54.17 | 20.65 |
|  |  | for women in the 18–40 year group | 7.69 | 6.51 | 20.71 | 2.37 | 3.55 | 46.74 | 12.43 |
|  |  | for men in the 40–49 year group | 1.28 | 4.81 | 8.49 | 3.13 | 1.44 | 57.69 | 23.16 |
|  |  | for women in the 40–49 year group | 3.16 | 3.95 | 21.34 | 3.16 | 3.95 | 49.02 | 15.42 |
|  |  | for men in the 50–59 year group | .53 | 2.79 | 7.85 | 1.6 | 1.33 | 60.24 | 25.66 |
|  |  | For women in the 50-59 year group | 0.56 | 3.39 | 20.90 | 1.69 | 0.56 | 53.13 | 19.77 |
|  |  | for men in the ≥ 60 year group | 1.58 | 2.77 | 11.07 | 0.79 | 1.98 | 55.72 | 26.09 |
|  |  | for women in the ≥60 year group | 1.09 | 0.54 | 10.87 | 0 | 2.72 | 56.52 | 28.26 |
|  | Xiao Tang | for men in the 20- to 40-year-old age group | 5.98 | 21.56 | 2 | 4.39 | .8 | 50.90 | 14.37 |
|  |  | for men in the 40–60-year-old age group | 2.81 | 13.04 | 3.07 | 4.22 | 2.05 | 51.79 | 23.02 |
|  |  | for women in the 20- to 40-year-old age group | 24.68 | 20.85 | 5.11 | 3.83 | 2.55 | 34.47 | 8.51 |
|  |  | for women in the 40- to 60-year-old age group | 8.65 | 14.05 | 11.90 | 4.86 | 3.78 | 45.41 | 11.35 |
|  | Lee-Ching Hwang | men | 2.27 | 1.29 | .81 | .93 | .58 | 86.33 | 6.87 |
|  |  | women | 4.82 | 1.55 | .85 | .95 | .01 | 86.67 | 4.21 |

Table 17) TP of “dyslipidemia & hyperglycemia” state to other states in studies

|  | | | No component | overweight/ obesity | hypertension | dyslipidemia | hyperglycemia | 2-component | ≥3 component (MS) |
| --- | --- | --- | --- | --- | --- | --- | --- | --- | --- |
| Dyslipidemia  and hyperglycemia | Xiaoxian Jia | for men in the 18–49 year group | 0 | 0 | 0 | 0 | 24.62 | 10.218 | 14.07 |
|  |  | for women in the 18–49 year group | 0 | 0 | 0 | 0 | 25.91 | 9.615 | 16.40 |
|  |  | for men in the ≥ 50 year group | 0 | 0 | 0 | 0 | 20.75 | 9.165 | 24.26 |
|  |  | for women in the ≥ 50 year group | 0 | 0 | 0 | 0 | 9.62 | 10.85 | 25.28 |
|  | Xiaoxiao Chen | for men in the 18–40 year group | 3.33 | 9.07 | 9.44 | 2.5 | .83 | 54.17 | 20.65 |
|  |  | for women in the 18–40 year group | 7.69 | 6.51 | 20.71 | 2.37 | 3.55 | 46.74 | 12.43 |
|  |  | for men in the 40–49 year group | 1.28 | 4.81 | 8.49 | 3.13 | 1.44 | 57.69 | 23.16 |
|  |  | for women in the 40–49 year group | 3.16 | 3.95 | 21.34 | 3.16 | 3.95 | 49.02 | 15.42 |
|  |  | for men in the 50–59 year group | .53 | 2.79 | 7.85 | 1.6 | 1.33 | 60.24 | 25.66 |
|  |  | For women in the 50-59 year group | 0.56 | 3.39 | 20.90 | 1.69 | 0.56 | 53.13 | 19.77 |
|  |  | for men in the ≥ 60 year group | 1.58 | 2.77 | 11.07 | 0.79 | 1.98 | 55.72 | 26.09 |
|  |  | for women in the ≥60 year group | 1.09 | 0.54 | 10.87 | 0 | 2.72 | 56.52 | 28.26 |
|  | Xiao Tang | for men in the 20- to 40-year-old age group | 5.98 | 21.56 | 2 | 4.39 | .8 | 50.90 | 14.37 |
|  |  | for men in the 40–60-year-old age group | 2.81 | 13.04 | 3.07 | 4.22 | 2.05 | 51.79 | 23.02 |
|  |  | for women in the 20- to 40-year-old age group | 24.68 | 20.85 | 5.11 | 3.83 | 2.55 | 34.47 | 8.51 |
|  |  | for women in the 40- to 60-year-old age group | 8.65 | 14.05 | 11.90 | 4.86 | 3.78 | 45.41 | 11.35 |
|  | Lee-Ching Hwang | men | 2.27 | 1.29 | .81 | .93 | .58 | 86.33 | 6.87 |
|  |  | women | 4.82 | 1.55 | .85 | .95 | .01 | 86.67 | 4.21 |

Table 18) TP of “MetS” state to other states in studies

|  | | | No component | overweight/ obesity | hypertension | dyslipidemia | hyperglycemia | 2-component | ≥3 component (MetS) |
| --- | --- | --- | --- | --- | --- | --- | --- | --- | --- |
| MetS | Xiaoxian Jia | for men in the 18–49 year group | 0 | 0 | 1.91 | 0 | .23 | 3.77 | *75.25* |
|  |  | for women in the 18–49 year group | 0 | 0 | 13.52 | 0 | 3.9 | 3.655 | *60.64* |
|  |  | for men in the ≥ 50 year group | 0 | 0 | .83 | 0 | .1 | 3.105 | *80.43* |
|  |  | for women in the ≥ 50 year group | 0 | 0 | 6.04 | 0 | 2.14 | 2.718 | *75.52* |
|  | Xiaoxiao Chen | for men in the 18–40 year group | 0 | 0 | 3.78 | 0 | 0 | 23.92 | *72.30* |
|  |  | for women in the 18–40 year group | 0 | 0 | 25 | 0 | 0 | 42.31 | *32.69* |
|  |  | for men in the 40–49 year group | 0 | 0 | 2.6 | 0 | .21 | 22.56 | *74.64* |
|  |  | for women in the 40–49 year group | 0 | 0 | 4.88 | 0 | 2.44 | 36.59 | *56.09* |
|  |  | for men in the 50–59 year group | 0 | 0 | 2.13 | 0 | .15 | 21.46 | *76.26* |
|  |  | For women in the 50-59 year group | 0 | 0 | 1.59 | 0 | 0.79 | 24.60 | 73.02 |
|  |  | for men in the ≥ 60 year group | 0 | 0 | 2.70 | 0 | 0.45 | 19.37 | 77.48 |
|  |  | for women in the ≥60 year group | 0 | 0 | 2.83 | 0 | 0.94 | 19.81 | 76.42 |
|  | Xiao Tang | for men in the 20- to 40-year-old age group | 1.81 | 10 | 4.55 | 1.82 | 0 | 29.09 | *52.73* |
|  |  | for men in the 40–60-year-old age group | 2.06 | 2.32 | 2.57 | 0 | .52 | 23.2 | *69.33* |
|  |  | for women in the 20- to 40-year-old age group | 14.29 | 4.76 | 4.76 | 4.76 | 0 | 20.63 | *50.80* |
|  |  | for women in the 40- to 60-year-old age group | 2.07 | 4.14 | 2.07 | 0 | 1.38 | 17.93 | *72.41* |
|  | Lee-Ching Hwang | men | .52 | .65 | .91 | .195 | .26 | 6.14 | *91.14* |
|  |  | women | .45 | .9 | .45 | .675 | .45 | 4.10 | *92.31* |
